# Graphene-Based Electrochemical Sensors for Psychoactive Drugs

**DOI:** 10.3390/nano12132250

**Published:** 2022-06-30

**Authors:** Ramin Boroujerdi, Richard Paul

**Affiliations:** Faculty of Science and Technology, Talbot Campus, Bournemouth University, Fern Barrow, Poole BH12 5BB, UK; rpaul@bournemouth.ac.uk

**Keywords:** electrochemical sensors, graphene, 2D materials, toxicology, forensic science, pharmaceutical biosensors

## Abstract

Sensors developed from nanomaterials are increasingly used in a variety of fields, from simple wearable or medical sensors to be used at home to monitor health, to more complicated sensors being used by border customs or aviation industries. In recent times, nanoparticle-based sensors have begun to revolutionize drug-detection techniques, mainly due to their affordability, ease of use and portability, compared to conventional chromatography techniques. Thin graphene layers provide a significantly high surface to weight ratio compared to other nanomaterials, a characteristic that has led to the design of more sensitive and reliable sensors. The exceptional properties of graphene coupled with its potential to be tuned to target specific molecules have made graphene-based sensors one of the most popular and well-researched sensing materials of the past two decades with applications in environmental monitoring, medical diagnostics, and industries. Here, we present a review of developments in the applications of graphene-based sensors in sensing drugs such as cocaine, morphine, methamphetamine, ketamine, tramadol and so forth in the past decade. We compare graphene sensors with other sensors developed from ultrathin two-dimensional materials, such as transition-metal dichalcogenides, hexagonal boron nitrate, and MXenes, to measure drugs directly and indirectly, in various samples.

## 1. Introduction

Over the years, two-dimensional (2D) materials have been deployed to monitor a vast range of analytes, across a variety of disciplines, but it can be argued that the properties of the 2D materials are of particular relevance to the detection of psychoactive drugs due to the common requirement for miniaturized systems that allow on-site, discreet drug monitoring. 

The requirement for accurate, portable drug testing is typically assumed to be of relevance to forensic work, but with potential drug analytes spanning legal, illegal and medical drug compounds the requirement is also very relevant to clinical settings and workplace drug testing, particularly for safety critical industries [1].

Conventional methods of drug analysis such as chromatography [2], immunochromatography [3], mass spectrometry (MS) [4], nuclear magnetic resonance (NMR) [5], ultraviolet–visible spectroscopy (UV-Vis) [6], Fourier-transform infrared spectroscopy (FTIR) and IR [7], surface-enhanced Raman spectroscopy (SERS) and Raman [8], circular dichroism (CD) spectroscopy [9], and spectrofluorimetry [10], which can offer accurate and precise results, often lack portability, and can suffer from lengthy run times, high costs and complicated operation. Thus, new methods capable of providing rapid, specific responses whilst being cost effective and easy to use are still required [11,12]. Next-generation 2D material-based sensors have the potential to replace these conventional methods in certain circumstances to avoid their drawbacks.

Here, we examine graphene and some of the 2D materials which have shown promising applications in the electrochemical sensing of recreational drugs and discuss recent progress made with regard to those sensors.

## 2. Graphene-Based Electrochemical Drug Sensors and Biosensors

Among the different nanostructures, two-dimensional graphene has drawn the most attention [13]. This unique nanomaterial is a thin layer with a tightly conjugated carbon-based honeycomb structure possessing sp^2^-bonded carbon atoms with π–π interactions [14,15,16]. The characteristics of such carbon-based materials including fast electron transfer, electrical and thermal conductivity, mechanical strength, biocompatibility, low density, and large surface area have caused the material to be of great interest to researchers over the past two decades [14,17,18,19]. Graphene has been widely used to develop various types of electrochemical sensors [14,20,21]. The addition of functional groups can enhance graphene characteristics in any desired way, from enhancing its stability to making it interact selectively only with the molecule of interest. Multi-functional graphene-based materials have been examined for the purpose of developing sensors over the years. From homogenous mixing with polymers or metal organic frameworks to doping, bonding or simply decorating graphene with metals or simple metallic nanoparticles, and from anchoring selective groups to the numerous functional groups (–OH, C–O–C, and –COOH), on graphene oxide, or directly forming a bond between reduced graphene oxide and another organic molecule through chemical reactions, researchers have utilized many methods over the years to achieve an improved sensor with higher sensitivity and selectivity than its competitors [14].

There are seven main categories of psychoactive drugs, narcotics, depressants, stimulants, hallucinogens, cannabis, anabolic steroids and inhalants, which are identified to be mainly used for nontherapeutic purposes [22]. While graphene-based electrochemical sensors have only been developed for some psychoactive drugs, there is great potential for the further development and application of miniaturized nanomaterial-based sensors for on-site and laboratory-based testing. Here, we examine some of the graphene-based drugs sensors which have been developed to measure depressant and stimulant drug levels in various matrices over the past decade.

### 2.1. Depressants

Illicit drugs based on their effects on the central nervous system can be classified into various groups, as explained earlier. Depressants reduce stimulation and slow down message transfer in the body by lowering brain activity [23]. Most of the reported graphene-based electrochemical sensors in recent years have focused on detecting this class of drugs and key examples are discussed below.

### 2.2. Traditional and Semisynthetic Opioids

Opioid receptors are expressed by various cells, but mainly central and peripheral neurons. Studies have defined three main types of opioid receptors in the central nervous system, the mu, delta, and kappa receptors [24]. Opioids are natural (opiates, e.g., morphine), semisynthetic (e.g., heroin) and synthetic (e.g., fentanyl) psychoactive drugs that exert their effects primarily via these opioid receptors [25]. Natural and semisynthetic opioids have very similar molecular structures, with small differences. 

**Figure 1:**
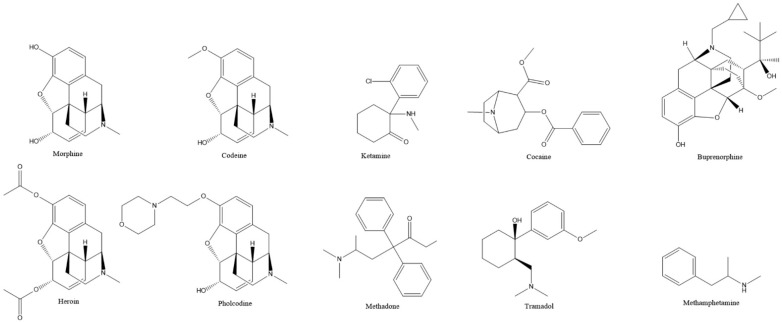
Molecular structure of the depressant and stimulant drugs studied in this review.

Several opioids share similar molecular structures to the popular pain medication morphine, often featuring a hydrophobic ring skeleton and a hydrophilic tertiary amine [26] (Figure 1), but despite these closely related structures, only four of these depressants have been studied with the help of graphene-based sensors: morphine, heroin, codeine and pholcodine.

Despite the similarities in their molecular structures, subtle differences in structure or changes in functional groups can affect drug potency. While morphine is an effective painkiller that is widely used, including for the long-term treatment of severe pain in cancer patients [27], its synthetic relative, pholcodine, behaves very differently in the body, and is used as an opioid cough suppressant with no analgesic or addictive properties [28].

When it comes to pharmaceutical and medical applications, the regular monitoring of these drugs is of the utmost importance as it can endanger the health of patients. At the same time, the accurate determination of morphine, codeine and heroin levels in biological samples is of vital interest in the control of drug abuse, clinical toxicology and forensic cases [12]. For instance, repeated or uncontrolled doses of morphine can cause side effects from nausea and vomiting to disruption in the central nervous system, toxication and death [29,30,31]. Considering the practical benefits of electrochemical sensors, including their affordability, ease of use and portability, graphene-based sensors have the potential to revolutionize drug-detection techniques.

The mechanism of the electrochemical detection of opioids with molecular structures similar to morphine has been linked to the electroactivity of both phenolic and tertiary amine groups in their structures. In the mechanism that includes phenol ring oxidation, each molecule loses a single proton which eventually causes the dimerization of morphine [32], whereas during the oxidation of the aliphatic tertiary amine group, two protons will be lost from morphine’s structure during the oxidation of the aliphatic tertiary amine group in morphine and end up etching the methyl group [32].

The very first study on graphene-based sensors for this class of chemicals was conducted in 2012, where a glassy carbon electrode was modified with graphene nanosheets and used to simultaneously measure morphine, heroin and noscapine simply based on their direct electrochemical oxidation. Three oxidation peaks appeared when a mixture of samples was tested, in the range between 0.3 and 0.9 V, where the first two belonged to morphine and noscapine and the third one represents the mixture of morphine and heroin. They used individual drug tests to identify the concentration of heroin at the third oxidation peak in the presence of the heroin and morphine mixture. They reported LoD values of 0.4, 0.5 and 0.2 μM for morphine, heroin and noscapine, respectively [33]. Since there are no drug-specific and selective receptors in the compartment of the pristine graphene sheets sensor, it is expected that it cannot work in complex matrices of real samples as it can generate an oxidation peak for other molecules in the sample environment.

Mohamed et al. were able to use 3D spongy adenine functionalized graphene as a sensor to measure paracetamol, codeine and caffeine simultaneously as each of these drugs developed a separate response (peak), at different potentials, in square wave voltammetry. A mixture of blood plasma and pain-relief tablets (Solpadeine) was used as an authentic sample, where the sensor was able to accurately measure the amount of added codeine in the real sample. The LoD of the sensor for codeine was reported to be 5.8 nM [34]. While graphene showed potential to sense codeine, Li et al. suggested that modifying a glassy carbon electrode with a graphene–nafion film can improve the electrical response (current) of the electrode to codeine when studying with cyclic voltammetry [35]. It is worth mentioning that nafion alone on a glassy carbon electrode also showed an oxidation peak in the presence of codeine, yet the intensity of the oxidation peak in the presence of graphene–nafion together was significantly higher.

Using a reduced graphene oxide–palladium hybrid as a selective modification for the glassy carbon electrode to sense morphine was studied in 2014. The electrode generated oxidation peaks related to the phenolic groups of morphine. The sensor was also tested with dopamine, uric acid, and ascorbic acid, showing separate oxidation peaks for each one of them, and then it was used to differentiate morphine oxidation peaks from the other three samples in a human urine test, which led to a detection limit of 40 nM for morphine [36]. However, the same as the pure graphene nanosheet sample, the sensor was not selective towards morphine only, yet the palladium seemed to improve the LoD of the sensor. At the same time, additional research was conducted on reduced multi-walled carbon nanotube-doped graphene oxide as a selective morphine detector. It has been reported that, as well as morphine, this sensor also showed oxidation peaks for dopamine and uric acid at different potentials, yet the presence of these chemicals did not affect the morphine oxidation peak intensity. It was also mentioned that the sensor did not respond to codeine, which shows a higher selectivity compared to previously developed graphene-based sensors; the LoD of the sensor was reported to be about 50 nM [37].

**Figure 2:**
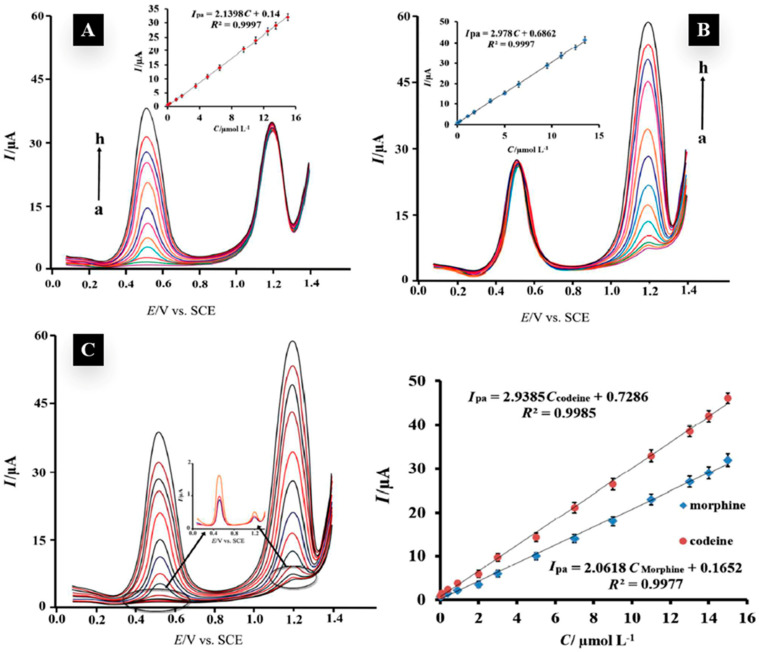
(**A**) DP voltammograms for morphine at the Zn_2_SnO_4_-GO/CPE in the presence of 10 μmol L-1 codeine in buffer solution (pH = 7.0); pulse amplitude = 50 mV and pulse time = 50 ms. (**B**) DP voltammograms for codeine at the Zn_2_SnO_4_-GO/CPE in the presence of 10 μmol L-1 morphine. (**C**) DP voltammograms for different concentrations of morphine and codeine in pH 7.0 B-R buffer solution. Adapted with permission from ref. [38]. Copyright 2016 RSC.

Bagheri et al. utilized a composite made of Zn_2_SnO_4_ nanoparticles with graphene in 2016. They achieved a homogenous composite through the hydrothermal reaction and heating a mixture of Zn_2_SnO_4_ nanoparticles and graphene oxide at 180 °C for 2 h. Despite the similarities between morphine and codeine, their sensor was able to generate two separate oxidation peaks for each one of the drugs and could simultaneously detect both drugs [38] (Figure 2). However, they did not study the selectivity of the sensor in the presence of any other chemicals. As composites made of metal oxides and graphene showed promising applications in sensing morphine, Beitollahi et al. reported a magnetic core–shell graphene oxide-Fe_3_O_4_@SiO_2_ composite as a morphine sensor with a linear range between 1 and 100 μM (LoD = 0.75 μM) using a differentiate pulse voltammetry system. They reported that the various sugars, salts and amino acids that were tested did not affect the response of the sensor to morphine, and ascorbic acid was the only interference they found; however, they suggested using ascorbic oxidase enzyme treatment to eliminate ascorbic acid in the samples and minimize its effects on the sensor [39].

Other than morphine, applications of graphene–metal oxide electrodes have also been studied for the drug codeine. Afkhami et al. reported a graphene–CoFe_2_O_4_ composite as a simultaneous detector of codeine and acetaminophen in urine, plasma, expectorant cold syrup and paracetamol tablets. The sensor showed two separate peaks for codeine (LoD = 0.011 μM) and acetaminophen (LoD = 0.025 μM) in square-wave voltammetry. While the sensor had not been tested in the presence of other common interferences such a morphine and ascorbic acid, it allowed them to measure codeine in various real samples, with reasonable accuracy and precision, close to the high-performance liquid chromatography (HPLC) results [40].

In 2019, Atta et al. attempted to improve morphine sensors using ionic liquids along with a reduced graphene oxide and cobalt oxide mixture. The authors formed different electrodes by mixing graphene, Co_3_O_4_, and various ionic liquids, and then modified each one of the electrodes further with the help of sodium dodecyl sulfate and tested them with urine samples and pharmaceutical morphine tablets to evaluate sensor performance. Similar to previous samples, sensors other than morphine still showed oxidation peaks for other molecules including ascorbic acid, dopamine and terazosin hydrochloride. The ionic liquid combination with sodium dodecyl sulfate increased the intensity of the oxidation peaks and helped with peak separation. The LoD of the sensor in the urine sample was found to be 0.484 nM [41]. Another study in the same year used long-carbon-chain cetyltrimethylammonium bromide molecules as a morphine-selective group. Abraham et al. applied graphene oxide-poly(CTAB) on a glassy carbon electrode to measure morphine with an LoD of about 0.36 μM; however, the sensor’s linear response range was between 50 and 60 μM [42]. The sensor showed separated oxidation peaks for ascorbic acid, uric acid and morphine, and they did not cover or overlap with morphine’s peak, yet the mixture of these two (ascorbic acid and uric acid) with morphine increased the electrical current response of the sensor compared to the response of the sensor to the same amounts of morphine without them.

A voltametric morphine immunosensor developed in 2017 based on gold-decorated graphene showed promise as a more sophisticated sensor. A gold-decorated graphene base was first covered with cysteamine and then morphine-selective antibodies were immobilized on the surface of the developed electrodes. This highly selective surface showed a selective range between 0.1 and 100 ng/mL, with a detection limit of 90 pg/mL [43]. Despite the high selectivity and sensitivity of such biosensors, they usually have drawbacks, including a short lifetime and sensitivity to temperature. Another biosensor that had significantly better selectivity compared to chemical sensors was developed from reduced graphene oxide-copper-poly(alanine). Copper chloride and alanine were left to react with each other in a buffer solution (pH = 7) to form a complex; then, through electropolymerization, the mixture formed a layer on the glassy carbon electrode, before graphene was added to the developed film with the same process. The obtained rGO-Cu-poly(Ala) sensor was able to detect morphine and diclofenac (LoD 47 nM) simultaneously in human blood samples, without any need to pretreat the authentic sample [44].

Reduced graphene oxide was also used to modify a membrane potentiometric pholcodine detector. The sensor includes the ion-association complex of pholcodine and 5-nitrobarbiturate, where o-nitrophenyloctyl ether worked as a solvent mediator and rGO was utilized as a solid contact ion-to-electron transducer in the compartment of the sensor. The sensor was tested on several drugs and salts (cations), and while similar molecules, including morphine, ethylmorphine and codeine, were found to have some interfering effects, the sensor was considered to be selective towards pholcodine. The LoD of the sensor was 0.04 μg/mL [28].

Nitrogen and sulfur co-doping has also been found to affect the charge densities around carbon atoms, resulting in an enhanced electrochemical sensitivity of graphene towards analytes such as morphine. It has been reported that using thiourea as a dopant on graphene, followed by chemical reflux and annealing, resulted in the co-doping of nitrogen and sulfur atoms on the surface of graphene at different weight percentages. Doped atoms affect the charge densities around carbon atoms, resulting in an enhanced electrochemical performance and detection of morphine. The authors suggested that increasing dopant concentrations prompts a higher electrocatalytic response towards morphine. Their co-doped graphene composite was used for the voltametric detection of morphine, which offered two separate linear ranges for higher concentrations (294–9.81 μg/mL) and low concentrations (4.59–0.574 μg/mL) and a LoD of 0.26 μg/mL. They successfully detected morphine in real soft drink, beer and vodka samples and suggested that the synergistic effect of the co-doping of N and S atoms on the graphene substrates was responsible for the sensitivity of their sensor [45].

### 2.3. Tramadol

Tramadol is a member of the phenanthrene opium alkaloids [46], and unlike standard opioid analgesics, tramadol eases pain through inhibiting the re-uptake of norepinephrine and serotonin and increasing their release [47,48,49]. This strong painkiller is widely used for the treatment of postoperative, neuropathic, somatic and visceral pain and so forth throughout the world [50,51,52]. While tramadol is considered a relatively safe pain medication when it is used in the prescribed dose, an overdose can have serious consequences, from nausea, vomiting and seizures to cardiopulmonary arrest and death [53,54,55].

Mohamed et al. compared the response of an unmodified carbon paste electrode (CPE) before and after the addition of a composite made of graphene oxide and multi-walled carbon nanotubes. They reported that the addition of GO-MWNTs improved the electroanalytical characteristics of the electrode and increased sensitivity towards tramadol, with a linear range between 2 nM and 1.1 mM [56]. This performance is directly related to the large surface area offered by graphene and carbon nanotubes. The combination of NiFe_2_O_4_ and graphene made one of the first nanocomposites that was used to measure tramadol electrochemically. The sensor was also able to measure acetaminophen as well, simultaneously in square-wave voltammetry. Metal oxide nanoparticles (NiFe2O_4_) were reported to enhance the sensitivity of the sensor, enabling a LoD of 3.6 nM for tramadol and 3 nM for acetaminophen. The sensor was tested to measure tramadol in urine, human serum, tramadol tablets and Ultracet tablets, where its measured concentrations matched the actual drug levels added in each sample and HPLC results [57]. Gold-decorated graphene also showed potential to detect and measure both tramadol and acetaminophen, simultaneously, in a composite with 4-hydroxy-2-(triphenylphosphonio) phenolate (HTP). The HTP modification went through oxidation and reduction in the presence of tramadol, which increases the peak current. The sensor was able to detect tramadol in urine and medicinal tablets by cyclic voltammetry. In this study, differential pulse voltammetry, due to it higher current sensitiveness comparing to cyclic voltammetry, was employed to measure the linear range and LoD, which were found to be 1–100 μM and 0.82 μM, respectively [58].

Further work in 2020 explored the applications of metal oxides for the detection of tramadol by linear sweep voltammetry, differential pulse voltammetry, cyclic voltammetry, and chronoamperometry. Decorated nanoparticles were obtained through a hydrothermal reaction, where cobalt salt and graphene oxide first went through a heating process in an autoclave in the presence of hydrogen peroxide and were washed with water, before going through another hydrothermal reaction in the presence of sodium borohydride, to finally form graphene–Co_3_O_4_. The developed sensor was able to detect tramadol in urine and in tablets with slight false positive error in a linear range between 0.1 and 500.0 μM, and with a LoD of 0.03 μM [59].

Bagheri et al. used silver-decorated graphene as the base for tramadol-selective molecularly imprinted polymers. The authors decorated graphene layers with silver using silver nitrate and sodium borohydride. The reducing agent (sodium borohydride) was responsible for reducing the graphene oxide and formation of silver nanoparticles. Molecularly imprinted polymers were developed by polymerizing a monomer in the presence of analyte and then the analyte was washed away to leave the developed selective frames ready to interact with the analyte in various samples. The sensor was casted using a paste made of decorated graphene; selective polymer frames and ionic liquids were used as conductive binder. The sensor was successful in accurately detecting tramadol in pharmaceutical tablets. The LoD of this potentiometric sensor was reported to be 2.09 nM [60].

A nanocomposite developed from graphene quantum dots modified with both praseodymium hydroxide nanoparticles and imidazolium ionic liquid has also been reported to be sensitive and selective towards low concentrations of tramadol. Cyclic voltammetry studies of tramadol with the developed electrochemical sensor showed the appearance of an irreversible anodic oxidation peak, where the intensity of the peak has a linear correlation with the concentration of tramadol in a wide range between 9 nM and 0.3 mM. The practical application of the sensor was tested and confirmed with tramadol medical injection vial and spiked blood serum. The detection limit of the sensor was found to be 3 nM. However, the electro-oxidation of tramadol and sensitivity of the sensor was reported to be highly affected by pH values [61].

### 2.4. Methadone and Buprenorphine

Methadone is a synthetic analgesic drug which has been utilized for treating opioid dependence for over half a century [62]. It has the potential to build up in the body to a toxic level if taken often or in high doses, or if it is used with some specific medicines and supplements [62]. Overdose with methadone can cause serious issues such as shallow breathing, stomach spasms, weak pulse, coma, and death [63,64]. In a recent study, a high-efficiency electrochemical sensor was developed by modifying glassy carbon electrode with silver-decorated graphene. Regarding the selectivity, the sensor was only tested in the presence of dopamine, ascorbic acid and uric acid, and these compounds caused less than 5% deviation in the response of the sensor. The LoD of the sensor was reported to be 0.12 μM [65]. However, other studies suggested that better detection limits for methadone can be achieved simply by changing the element decorated on the graphene. Nazari and Eshaghi decorated graphene oxide with thioglycolic acid (TGA)-bonded cadmium selenide quantum dots (CdSe QDs). A functionalized graphene layer with core–shell QDs was able to detect morphine and methadone with the detection limits of 0.03 μM and 0.04 μM [66], respectively, which is more than three times better than what was previously achieved by silver decoration [65].

Buprenorphine is a narcotic analgesic that acts similarly to methadone in terms of its action and utilization [67]. In a similar manner to tramadol, buprenorphine is also used as a pain killer and for detoxification in patients with moderate levels of opioid dependence [50]. Buprenorphine is also a strong synthetic pain killer which is used to treat addiction to opioids [68]. Fakhari et al. explored the addition of graphene to electrodes to improve the detection of buprenorphine, and reported that graphene addition improved the sensitivity of the sensor to submicromolar detection limits (0.16 μM) through differential pulse voltammetry [67]. They also used cyclic voltammetry in combination with modeling (density functional theory) to investigate buprenorphine’s oxidation pathway and they reported three oxidation peaks at about +0.4, +0.8 and +1.1 V, which are related to the oxidation of the phenolic group, pseudo-buprenorphine and the amino group, respectively.

### 2.5. Ketamine

Ketamine is another analgesic drug with wide application in the medical field [69,70]. However, the abuse of ketamine is increasing to the point that it has become a major concern, not only due to its abuse for recreational purposes [71,72], but also because this colorless, odorless and tasteless drug facilitates sexual assaults when it is added to drinks [73,74]. Miniaturized nanomaterial-based sensors could provide fast, portable detection, to help monitor drug abuse in any situation.

**Figure 3:**
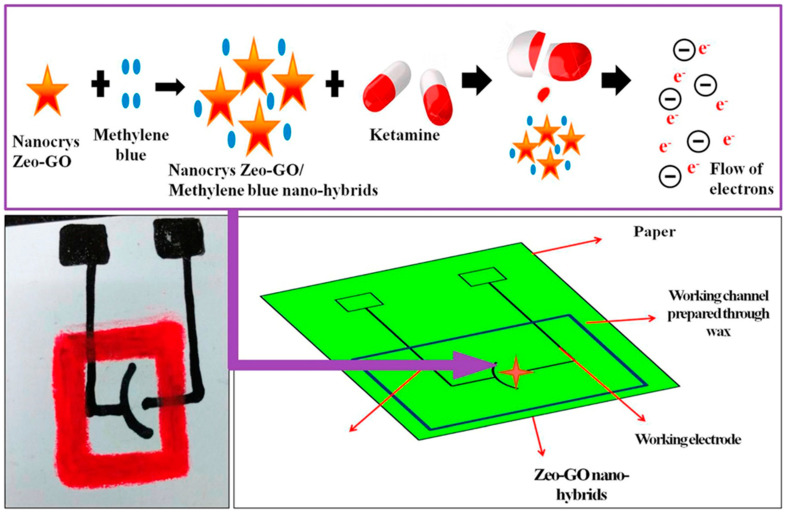
Schematic representation of the fabrication of the working paper-based electrode and the sensing mechanism of ketamine. Adapted with permission from ref. [75]. Copyright 2017 ELSEVIER.

The idea of using a microfluidic paper-based analytical device with a chip developed from zeolites nanoflakes and graphene oxide (Zeo–GO) was one of the first studies in the development of a graphene-based electrochemical sensor for this date rape drug that achieved a detection limit of 1 nM and showed application in both alcoholic (whisky) and nonalcoholic (juice) drinks (Figure 3). The use of a paper chip reduced the production costs of the electrode, while Zeo-GO helped with the amplification of the generated signal in the presence of ketamine in the cyclic voltammetry system. Methylene blue was also used in the sensing environment to help with the electro-oxidation reaction of (reducing) ketamine [75]. The sensor was reported to be selective, with ascorbic acid as the only possible significant interference among the tested cations, sugars and amino acids, though the sensor was not tested with any other drugs.

Molecularly imprinted polymers have also been used for ketamine-selective functionalization for graphene-based sensors. In 2019, Fu et al. suggested using a molecularly imprinted membrane on a graphene layer which was previously modified with metal–organic frameworks in compartments of an electrochemical sensor to detect and measure ketamine levels in biological fluids. Firstly, they developed a metal–organic framework (MOF) by a solvothermal reaction using 1,4-benzendicarboxylicacid and zirconium metal salt. A paste was made of graphene and Zr-MOF made in ethanol and used to cast the base of the sensor. To make the selective membrane, monomers were mixed with ketamine and the mixture was dropped onto the surface of the casted electrode to form a thin layer. The polymerization of monomers started when the developed surface was exposed to UV light, and ketamine molecules were later simply washed from the polymer membrane surface with methanol and acetic acid. Cyclic voltammetry was utilized to measure the response of the electrode to ketamine in various samples. The sensor showed selective characteristics towards ketamine and its metabolite norketamine, as it did not respond to methylenedioxymethamphetamine, methylamphetamine, dopamine, and ascorbic acid. The selective membrane let the electrode selectively and accurately measure ketamine in urine and saliva. The linear range of the sensor was 0.1 nM to 0.4 μM, with a LoD of about 0.04 nM [76].

### 2.6. Stimulants

Stimulants function in a manner opposite to depressant drugs and mainly accelerate brain activity. They increase the mood of the user and make them feel energetic. This class of drugs are highly reinforcing, as they directly affect brain systems implicated in motivated behavior, such as the basal ganglia and the limbic system, and they modulate control systems in the prefrontal cortex [77,78,79].

### 2.7. Cocaine

Cocaine and amphetamines are among the most abused and powerful stimulants [23], and both of them are among the very few stimulants that have been detected with the help of graphene-based electrochemical sensors.

Cocaine has strong potential for addiction and illegal abuse. The sensitive and accurate detection of cocaine in various samples is therefore of critical importance, not only for clinical diagnostics, but also for legal and forensic investigations [80,81,82]. Despite the fact that cocaine is the second most consumed illicit drug in Europe (after cannabis) [83], and despite the benefits of graphene-based sensors [14], not many papers have focused on developing a graphene-based sensor for this compound.

**Figure 4:**
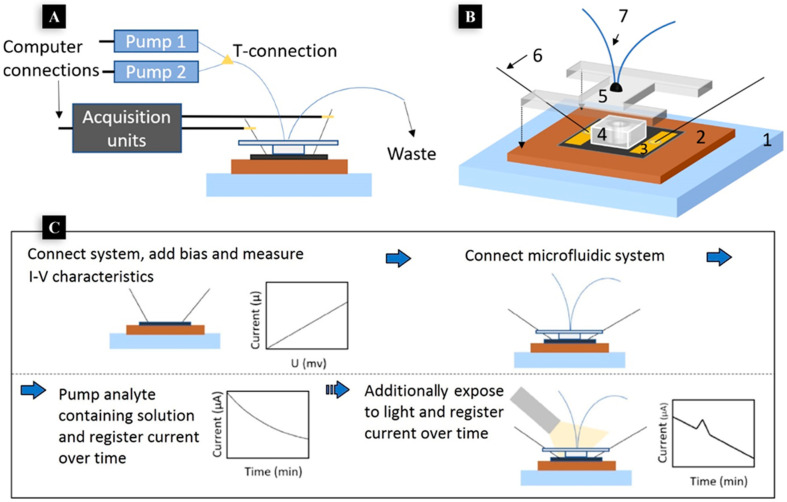
(**A**,**B**), at the top, show an overview of the setup and all connections and the schematic of the setup with all essential components marked. 1: stage; 2: holder; 3: fabricated graphene-based device; 4: PDMS-based flow chamber; 5: PMMA lid; 6: needle pin probes; 7: microfluidic tubing. General scheme for the detection of different analytes with the fabricated graphene-based device including additional exposure to light to investigate the discovered photo-physical response illustrated in the box at the bottom (**C**). Adapted with permission from ref. [85]. Copyright 2019 MDPI.

While a few papers studied the applications of graphene for developing a selective fluorescence sensor to measure cocaine [80,84], Karlsson and his team were able to introduce a graphene-based electrochemical biosensor in 2019. Their detection system still relies on light-beam responses, though they used a thin layer of graphene on SiC substrate as an electrochemical sensing platform to detect cocaine and amphetamine. Transmission line measurements were used in the compartment of the developed sensor to constantly monitor and measure the changes in resistance of the fabricated graphene-based surface. They immobilized drug-selective antigens (anti-cocaine and anti-amphetamine) on the surface of graphene with the help of 1-pyrene butyric acid-N-hydroxy-succinimide ester (pyrene-NHS) and used a flow system to pump analytes over the active sensing area and studied the changes in the electrical current over time. The samples were pumped to the surface of electrode, and a light source was used to cause a photophysical response, in a way that only in the presence of molecules of interest did light cause a spike (peak) in the recorded electrical current (Figure 4). The authors suggested that the same technique could be used to measure other drugs, but the antibody itself does not show photoactivity, so any photoactivity and changes in the electrical current can be related to the analyte [85].

Another cocaine-selective electrode was developed by utilizing molecularly imprinted polymers on the surface of palladium-decorated graphene. While codeine itself was used as the template molecule to develop selective p-aminobenzoic-acid-based polymer frames through electrodeposition, palladium decoration was applied to enhance the communication between the imprinted polymer and graphene. Square-wave voltammetry was used for cocaine quantification with this sensor, and showed a linear response in the range of 100–500 µM, with a detection limit of 50 µM. Real street samples of cocaine, saliva (spiked cocaine) and river water (spiked cocaine) were studied as authentic samples and the sensor was able to detect and measure drug levels close to the spiked levels [86].

Rocha et al. developed a 3D-printed graphene–polylactic acid electrode to measure low concentrations of cocaine in various samples with a simple sample pretreatment step (electrolyte addition) by utilizing square-wave voltammetry [87]. They used graphene–polylactic acid filament to 3D print a working electrode; this sensor could detect cocaine with a LoD of 6μM with square-wave voltammetry. Despite introducing a novel application and the affordability of the developed cocaine sensor, this method and electrode were previously developed and reported by Cardoso et al., who used it to measure phenolic compounds such as dopamine [88], so the selectivity of the sensor might be affected by the presence of complex matrices. Rocha et al.’s 3D electrode for cocaine detection was reported to respond to various compounds at different potentials in square-wave voltammetry; however, the oxidation peak belonging to cocaine only appeared at a single potential, even in the presence of other chemicals in the environment, which could suggest the possibility of the simultaneous detection of different chemicals [87].

### 2.8. Methamphetamine

Methamphetamine is another powerful central nervous system (CNS) stimulant that is widely abused. This drug also has medicinal applications such as the treatment of patients with obesity or drinking problems [89]. Its uncontrolled use can affect body balance, cause increased blood pressure and heart rate, and affects body temperature and behavior [90,91]. Many authors have, in the past, worked on voltammetric, potentiometric, impedimetric, amperometric and electrochemiluminometric sensors to detect methamphetamine [92]; there is less research utilizing graphene’s unique characteristics to develop a selective graphene-based electrochemical sensor for methamphetamine.

**Figure 5:**
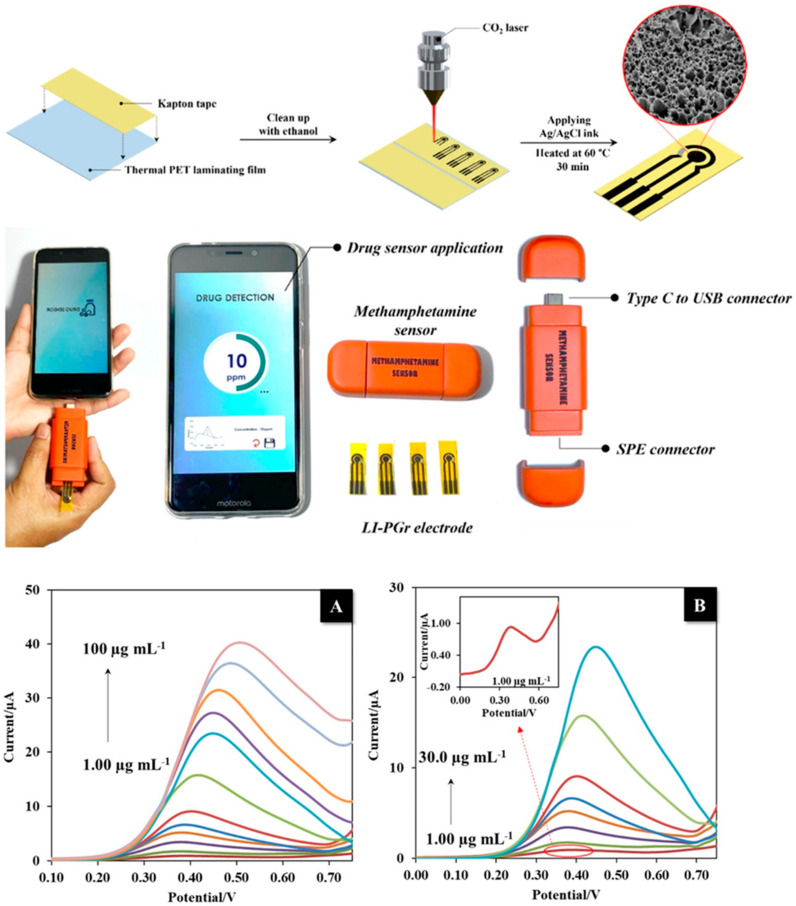
Schematic representation of the laser-induced porous graphene electrode fabrication and the components of the developed portable methamphetamine sensor illustrated at the top of the figure. (**A**,**B**) show the analytical characteristics of the sensor: (**A**) DPV responses of MA in the developed portable device. (**B**) DPV responses of MA, while showing inset—amplified anodic peak current of MA at a concentration of 1.00 µg mL^−1^. Adapted with permission from ref. [93]. Copyright 2022 MDPI.

A simple and affordable graphene-based methamphetamine sensor was developed using a simple, one-step fabrication method by Saisahas et al. In their method, they placed Kapton tape on polyethylene terephthalate substrate and used a CO_2_ laser to induce 3D porous graphene on it. The method was solvent free and allowed them to fabricate various designs. Their flexible sensor was then tested with a portable potentiometer in a cyclic voltammetry system, where it was shown to have a LoD of 0.3 μg/mL. To investigate selectivity, they tested the sensor’s response versus sugars, salts (cations and anions), uric acid, urea and ascorbic acid, and they reported a highly selective response for methamphetamine (Figure 5). Regarding the real samples, they tested the sensor with saliva and household surfaces (sample recovered from glass, stainless steel and plastic surfaces), where they suggested that the satisfactory recovery results of testing real samples can indicate that their sensor has the potential to be applied in forensic investigations [93].

**Figure 6:**
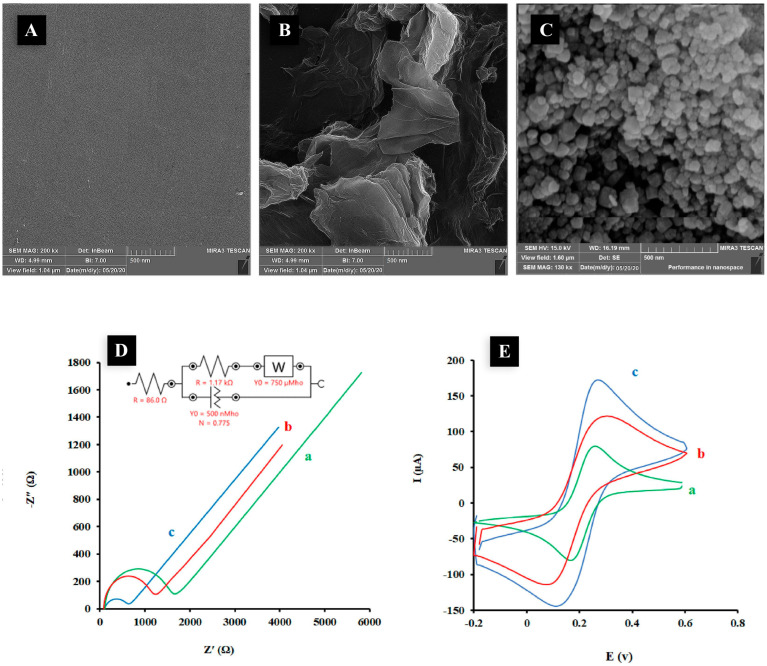
The SEM images of the surface of bare GCE (**A**), ErGO/GCE (**B**), and Fe_3_O_4_@PPy/ErGO/GCE (**C**). The Nyquist plots (**D**) and CVs (**E**) (recorded at scan rate of 50 mV/s) of bare GC (curve a), ErGO/GC (curve b) and Fe_3_O_4_@PPy/ErGO/GC (curve c) in solution containing KCl (0.1 M) and Fe(CN)63-/4- (5 mM). EIS conditions: initial potential, 0.17 V; frequency range, 100 kHz to 0.1 Hz. Adapted with permission from ref. [94]. Copyright 2021 ELSEVIER.

Another methamphetamine-selective electrochemical sensor fabricated by Riahifar et al. also used reduced graphene oxide as the base of the sensor, but with further functionalization. First, graphene oxide was reduced on the surface of a glassy carbon electrode. Then, iron chloride was turned to iron oxide in the presence of ammonium hydroxide, followed by polymerization in a mixture with pyrrol monomers, resulting in conductive polypyrrol forming a shell around the Fe_3_O_4_. The core–shell nanoparticle suspension was then added to the surface of the rGO drop by drop and left to dry. A urine sample and pretreated human blood serum were used as real samples for this study, where 1 mL blood was mixed with 9 mL ethanol and vortexed for 10 min, and the obtained clear layer was separated and diluted 10 times with buffer (pH = 8) to be analyzed with cyclic voltammetry. The LoD of the sensor was found to be 1 nM with a linear range between 0.005 and 200 μM. The selectivity of the sensor was not studied compared to other drugs, though the sensor did not respond to tested amino acids and salts [94] (Figure 6). In a similar context, Anvari et al. tried to use graphene and metal oxide on a glassy carbon electrode to detect methamphetamine; however, instead of adding graphene and metal oxide nanoparticles separately, as different layers, they made a graphene–metal oxide composite first. Cerium nitrate was turned to cerium oxide by ammonium hydroxide, and then graphene oxide was added to it and reduced in the presence of hydrazine. Ce_2_O_3_-decorated graphene was then placed and dried on the surface of the glassy carbon electrode. Samples were studied with this electrode using square-wave voltammetry. The addition of drugs such as acetaminophen, morphine, tramadol and cocaine, metal salts, uric acid, dopamine, glucose, and ascorbic acid had an insignificant effect on the response of the sensor to methamphetamine at the studied peak potential. The minimum concentration of methamphetamine that the sensor was able to detect was 8.7 μM [95].

As can be concluded from Table 1, graphene plays an essential role in almost all 2D drug sensors and can act as a base to hold other nanoparticles as well. The superior properties of graphene-based materials were exploited for the sensing of drugs and alcohols mostly in composite rather than pristine form. Only a few studies have considered the sensing behavior of pure graphene or graphene oxide against drugs [33,67]. Table 1 shows that most researchers preferred to use secondary nanoparticles as receptors over the graphene base, where most of the studies used the exact same procedure, but adapted to the reagent choice to improve the detection limit. For example, the detection of acetaminophen was achieved with the help of both NiFe_2_O_4_ and CoFe_2_O_4_ [40,57], and, as can be seen, the only difference here was switching the nickel and copper to achieve better detection limits; additionally, it can be seen that noble metals such as Ag-NPs, Pd-NPs and Au-NPs have been used for the detection of various drugs, mainly cocaine, in various papers [96,97]. While these papers provide useful indications as to the sort of compounds that may be successful in detecting certain drugs when used as a decoration on graphene, it is clear that more studies on the use of functionalized graphene with a direct anchoring of selective groups on the graphene surface are needed.

## 3. Other 2D Materials as Electrochemical Drug Sensors

While two-dimensional graphene, due to its large surface area, mechanical strength, affordability, and high conductivity, is considered to be one of the best available options to develop electrochemical sensors, it is not the only known ultrathin material. Scholars have developed similar 2D materials with large surface areas from other compounds and applied them in the compartments of sensors and biosensors [14]. Though not many of these sensors have been used to measure recreational drugs directly, they show promising applications in sensing neurotransmitters, which allows researchers to investigate indirect drug measurements with the help of such biomolecules. Dopamine is one of these neurotransmitters, which is important in bioanalytical and forensic science studies because it could be used to investigate drug usage indirectly, since its production in the body is increased following the use of recreational drugs [98]. As a significant neurotransmitter in the human central nervous and hormone system, dopamine participates in many physiological processes but is mainly involved in behavioral responses and the release of other hormones [99,100].

Thin, layered materials, similar to graphene, that have been reported and studied for their applications in developing electrochemical sensors to measure dopamine include transition metal dichalcogenides (TMDs), MXenes, hexagonal boron nitride (*h*-BN) and very thin layers of black phosphorus and phosphorene. First, we briefly review their characteristics before discussing their sensing applications.

### Characteristics and Applications of Other 2D Materials for Sensing Purposes

Transition metal-based 2D materials can be categorized into five subcategories based on their molecular structures (Table 2). They can be described from layers with MX molecular structure (M is the transition metal) up to M_4_X_3_ structures [101,102,103,104]. Table 2 illustrates the difference between various groups of transition metal-based 2D materials. Transition metal halides (TMHs) include a group of materials with MY_2_ and MY_3_ formulas, with halogen elements (Y) instead of common elements such as carbon, nitrogen, etc. (X) [105,106]. While TMDs benefit from a remarkably large surface area, high electrical conductivity and variable oxidation states [107], ultrathin MXenes layers also benefit from the metallic conductivity of transition metal carbides and offer a relatively large surface [108]; these characteristics provide the basis for their use as sensitive electrochemical sensors. Two-dimensional hexagonal boron nitride (*h*-BN) has shown excellent electrocatalytic activity, offering active nitrogen and boron at edges, defects similar to graphene, and an adjustable band gap which can be modified with the help of other nanoparticles or chemical functionalization. *h*-BN has proved to be another ideal thin material for electrochemical sensing applications [109].

The pursuit for the discovery and development of 2D materials, similar to graphene, caused rapid growth on both theoretical and experimental fronts, which led to the recent discovery of a single 2D layer of BP called phosphorene [110,111] in 2014, which has numerous foreseeable applications [112,113]. BP is known as a semiconductor and its band gap increases when it forms thinner layers (band gap of about 0.3 eV for bulk and about 2 eV for single-layer phosphorene) [114,115]. A hole mobility of 300–1000 cm^2^V^−1^s^−1^ was obtained from the field effect transistors based on phosphorene on Si, which suggested that it could be further enhanced to 2000–4000 cm^2^V^−1^s^−1^ when it is located on the *h*-BN substrate or between two *h*-BN flakes at low temperature [111,116,117,118,119]. The high conductivity of these materials also suggests promising applications in the compartments of electrochemical sensors. The cytotoxicity of BP nanomaterials was preliminarily studied and showed no observable toxicity in various cells [120,121,122,123] and supports the idea that it can be used to develop safe biosensors. As an example, the combination of a large surface area and high electrical conductivity leads to the development of a BP-based biosensor applied for the detection of trace human immunoglobulin G (IgG) with a detection limit of 10 ng/mL and fast response time in the order of seconds [124].

**Table 3:** Comparison of the LoD of ultrathin material-based sensors and biosensors.

Analyte	2D Base Material	Sensing Materials	Limit of Detection	Ref.
Dopamine	Graphene	G-C_3_N_4_/CuO	0.1 nM	[100]
		rGO-poly(Cu-AMT)	3.48 nM	[125]
		PEDOT/GO/in vivo carbon fiber	85 nM	[126]
	TMDs	MoSe_2_/Graphene	1 nM	[127]
		MoS_2_	1 µM	[128]
	MXenes	Ti_3_C_2_T_x_/Pt-NPs	6 nM	[129]
		Ti_3_C_2_T_x_	100 nM	[130]
	*h*-BN	MIP/G-QDs/h-BN	20 pM	[131]
	BP	BP	NA	[132]

As expected from 2D-based sensors, (in Table 3), we can see how 2D materials offer detection limits in the nanomolar region concentration, and even in the case of *h*-BN-based sensors the LoD can be as low as 2 × 10^−13^ molar [131]. TMDs and BP show selective behavior as well, but the modification of the material can lead to improved sensitivity and lower detection limits.

## 4. Comparison of Electrochemical Sensing and Other Conventional Drug-Detection Techniques

Traditionally, the majority of drug-detection methods using analytical systems are undertaken using chromatography-based techniques (gas chromatography (GC) and HPLC), spectroscopy techniques (refractive index (RI), UV-Vis, fluorescence, evaporative light scattering, and NMR), or hyphenated approaches (GC-MS and HPLC-MS). Each of these techniques has their own advantages and disadvantages, but among them, three systems are more popular, especially when it comes to drug analysis: UV, fluorescence and MS [133,134,135,136,137,138,139,140,141]. While such techniques have proved to be reliable and allow the analysis and detection of almost all molecules of interest in various samples, they still have unsolvable issues. Samples typically must be collected remotely and transferred to the lab, and go through a pretreatment process before they can be introduced to the system for analysis. This step alone can lead to a loss of or reduction in many drugs through degradation or inefficient extraction. In addition, sample preparation steps and the required data interpretation following analysis can be technically challenging and require expertise. Comparing these techniques with point of care (POC), portable devices, which can analyze samples on the spot, can provide results in the form of a simple line or number, which are usually simple enough to be read and understood with basic training. The issue with most POC devices is usually the low sensitivity and lack of accuracy. The sufficient miniaturization of systems (so as to make them truly portable, i.e., handheld) such as GCMS is highly challenging and not yet a reality, so the alternative is to improve the sensitivity of portable sensor devices. There are a range of sensitive portable electrochemical sensors, such as blood sugar sensors, but only a few have been developed to detect and measure drugs or toxins accurately and sensitively. Due to the large surface area of nanoparticles, not only they can reduce the cost of developing a sensitive system, but they can also help with developing much more sensitive and selective portable sensors to work in a variety of samples. The analytical variables of some of the introduced sensors are compared with HPLC and GC techniques in Table 4. As can be seen in said table, graphene-based electrochemical sensors not only can compete with chromatography techniques in terms of detection limits, but they can also provide results in a significantly shorter period of time.

## 5. Conclusions and Outlook

Graphene or graphene oxide have been applied as sensitive drug sensors, often without significant functionalization, due to the ability of each molecule to form oxidation or reduction peaks when studied with cyclic or square-wave voltammetry. Whilst many studies have reported such applications, it is apparent that for the technique to become more commercially viable the selectivity of these sensors needs further demonstration. 

Many of the reportedly selective drug sensors have only been tested against simple cations, anions, amino acids and small biological molecules, and so it is not clear how those sensors would respond in the presence of other drugs or large molecules, especially as many of the drugs often have similar molecular structures. In the case of monitoring street drugs, such compounds usually do not have high purity and there is a need to monitor drugs on potentially contaminated surfaces and in complex matrices such as biological fluids. The papers reviewed here clearly demonstrate the potential of graphene to be used in the development of selective electrochemical sensors for drug targets, but the selectivity of sensors needs to improve further as the reliability of sensors with this application is a must for pharmaceutical and forensic monitoring.

Graphene alone has been shown to improve the sensitivity of sensors and the decoration of graphene with metal or metal oxides can improve sensitivity even further. The development of selective receptors seems to be challenging and biosensors seem to be one of the most selective options available. However, due to the difficulties associated with biosensors, such as their short lifetimes, a viable alternative may be molecularly imprinted sensors. A promising area for this field to develop into may be the development of chemical functional groups, anchored to the surface of graphene, to interact with large drug molecules. This is currently an under-researched area of graphene-based sensors. Anchoring functional groups to graphene, instead of using of polymers, has the potential to improve the conductivity, resulting in faster, more sensitive drug sensors. 

As expected, and illustrated in some of the discussed papers, CV, SWV and DPV were the most common methods of studying the behavior of electrochemical sensors; pulse techniques, such as DPV, are more sensitive than linear sweep methods due to the minimization of the capacitive current and sensors were able to detect and measure even smaller changes.

Graphene-based electrochemical sensors targeting depressants such as morphine have been widely studied, but the use of graphene in the detection of other classes of drugs such as stimulants or hallucinogens, and especially the recent focus on novel psychoactive substances, is a less studied field, with high potential for publication. If we consider the potential of other 2D materials for application as drug sensors, the potential is equally high. The large surface area and high electrical conductivity of such materials provides excellent possibilities for future development in this field, with real potential for academic and commercial impacts.

## Figures and Tables

**Table 1:** Comparison of functionalizations, method of measurement and LoD of graphene-based electrochemical sensors for the determination of drugs in various samples.

NO	Drug(s) Detected	Sensing Material	Main Method(s)	Detection Limit(s)	Sample(s)	Ref.
*1*	Morphine	Graphene oxide–Co_3_O_4_	DPV/CV	0.54 nM	Urine, Medical tablets	[41]
*2*	Morphine	Graphene–Pd NPs	DPV	12.95 nM	Urine	[36]
*3*	Morphine	Graphene–Cu-poly(Ala)	DPV	47 nM	Blood serum	[44]
*4*	Morphine	Graphene-MWCNTs	CV	50 nM	Urine	[37]
*5*	Morphine	Graphene oxide–poly(CTAB)	DPV/CV	360 nM	Blood serum, Urine	[42]
*6*	Morphine	Graphene oxide–Fe_3_O_4_@SiO_2_	DPV/CV	750 nM	Urine, Medical ampoule	[39]
*7*	Morphine	Graphene–Au NPs-MO antibody	SWV	90 pg/mL	Saliva	[43]
*8*	Morphine	N-S-doped graphene	CV	0.26 μg/mL	Soft drinks, Beer, Vodka	[45]
*9*	Heroin, Morphine, Noscapine	Graphene	DPV	500, 400 and 200 nM	None	[33]
*10*	Morphine and Codeine	Graphene–Zn_2_SO_4_	DPV/CV	11 and 9 nM	Urine, Plasma, Medical tablets	[38]
*11*	Codeine	3D spongy graphene–adenine	SWV	5.8 nM	Plasma, Solpadeine tablets	[34]
*12*	Codeine	Graphene–Nafion	SWV	15 nM	Urine, Cough syrup	[35]
*13*	Codeine and Acetaminophen	Graphene–CoFe_2_O_4_	SWV	25 and 11 nM	Plasma, Urine, Medical tablets, Expectorant Cod syrup	[40]
*14*	pholcodine	Graphene–(PHL)^2+^-(NB)^−^	POT	0.04 μg/mL	Medical suspensions, Medical syrups	[28]
*15*	Ketamine	Graphene oxide–Zeo	CV	0.001 nM	Whisky, Juice	[75]
*16*	Ketamine and Norketamine	Graphene–MOF-MIM	DPV	0.04 nM	Urine, Saliva	[76]
*17*	Tramadol	Graphene–Ag NPs-MIP	CV	2.04 nM	Urine, Medical tablets	[60]
*18*	Tramadol	1-M-3-BBr-Pr(OH)_3_-GQD	CV	3 nM	Medical injection, Blood serum	[61]
*19*	Tramadol	Graphene oxide–MWCNTs	CV	15 nM	Plasma, Medical tablets	[56]
*20*	Tramadol	Graphene–Co_3_O_4_	LSV/DPV	30 nM	Urine, Medical tablets	[59]
*21*	Tramadol and Acetaminophen	Graphene–NiFe_2_O_4_	SWV	3.6 and 3 nM	Blood serum, Urine, Medical tablets, Ultracet tablet	[57]
*22*	Tramadol and Acetaminophen	Graphene–Au NPs-HTP	DPV	820 nM	Urine, Medical tablets	[58]
*23*	Methadone	Graphene–Ag NPs	DPV	120 nM	Blood serum	[65]
*24*	Methadone and Morphine	Graphene–TGA@CdSe	DPV	40 nM and 30 nM	Blood serum	[66]
*25*	Buprenorphine	Graphene	DPV/CV	160 nM	Urine	[67]
*26*	Cocaine	3D printed Graphene–PLA	SWV/CV	6 μM	None	[87]
*27*	Cocaine	Graphene–Pd NPs-MIP	SWV	50 μM	Saliva, River water, street sample	[86]
*28*	Cocaine and Amphetamine	Graphene–(pyrene-NHS)-antibody	TLM	NA	None	[85]
*29*	Methamphetamine	Graphene–Fe_3_O_4_@PPy	CV/SWV	1 nM	Blood serum, Urine	[94]
*30*	Methamphetamine	Graphene–Ce_2_O_3_	SWV	8.75 μM	Plasma	[95]
*31*	Methamphetamine	3D Porous Graphene	DPV/CV	0.31 μg/mL	Saliva, Household surfaces	[93]

**Table 2:** Atlas of 2D materials based on metals and transition metals.

Class of 2D Material	Mono-Layer Molecular Structure	Possible “M/M’”s	Possible “X/Y”s
Semimetal Chalcogenides (MCs)	M’X	Ga and In	S, Se and Te
Transition Metal Chalcogenides (TMCs)	MX	Transition metals between group 4 and group 10	
	M_3_X_4_		
Transition Metal Dichalcogenides (TMDs)	MX_2_		
Transition Metal Halides (TMHs)	MY_2_		Cl, Br and I
	MY_3_		
Transition Metal Carbides, Nitrides and Carbonitrides (MXenes)	M_2_X		C and N
	M_3_X_2_		
	M_4_X_3_		

**Table 4:** Comparison of electrochemical sensors and chromatography techniques.

Analyte	Experimental Technique	Duration of Analysis ^a^	Detection Limit	Ref.
Morphine	HPLC-UV	10 min	1 ng/mL	[142]
	GC-MS	11 min	3 ng/mL	[143]
	CV	24 s ^b^	0.54 nM	[41]
Tramadol	HPLC-UV	40 min	6.7 ng/mL	[144]
	GC-MS	9 min	0.77 ng/mL	[145]
	CV	3.4 s ^b^	2.04 nM	[60]
Cocaine	HPLC-Fluorescence	8 min	1 ng/mL	[146]
	GC-MS	16 min	0.05 ng/mL	[147]
	CV	60 s ^b^	6 μM	[87]
Methamphetamine	HPLC-MS	12 min	0.05 ng/mL	[148]
	GC-MS	18 min	0.06 μg/mL	[149]
	CV	40 s ^b^	1 nM	[94]

^a^: Run time—the pretreatment process time of samples excluded. ^b^: Full CV cycle at studied scan rate—in case of single potential measurements, it will take less than 1 s.
